# SEdb 2.0: a comprehensive super-enhancer database of human and mouse

**DOI:** 10.1093/nar/gkac968

**Published:** 2022-11-01

**Authors:** Yuezhu Wang, Chao Song, Jun Zhao, Yuexin Zhang, Xilong Zhao, Chenchen Feng, Guorui Zhang, Jiang Zhu, Fan Wang, Fengcui Qian, Liwei Zhou, Jian Zhang, Xuefeng Bai, Bo Ai, Xinyu Liu, Qiuyu Wang, Chunquan Li

**Affiliations:** The First Affiliated Hospital, Institute of Cardiovascular Disease, Hengyang Medical School, University of South China, Hengyang, Hunan 421001, China; School of Medical Informatics, Daqing Campus, Harbin Medical University, Daqing 163319, China; The First Affiliated Hospital, Institute of Cardiovascular Disease, Hengyang Medical School, University of South China, Hengyang, Hunan 421001, China; School of Computer, University of South China, Hengyang, Hunan 421001, China; The First Affiliated Hospital, Cardiovascular Lab of Big Data and Imaging Artificial Intelligence, Hengyang Medical School, University of South China, Hengyang, Hunan 421001, China; The First Affiliated Hospital, Institute of Cardiovascular Disease, Hengyang Medical School, University of South China, Hengyang, Hunan 421001, China; School of Medical Informatics, Daqing Campus, Harbin Medical University, Daqing 163319, China; The First Affiliated Hospital, Institute of Cardiovascular Disease, Hengyang Medical School, University of South China, Hengyang, Hunan 421001, China; School of Computer, University of South China, Hengyang, Hunan 421001, China; The First Affiliated Hospital, Cardiovascular Lab of Big Data and Imaging Artificial Intelligence, Hengyang Medical School, University of South China, Hengyang, Hunan 421001, China; School of Medical Informatics, Daqing Campus, Harbin Medical University, Daqing 163319, China; School of Medical Informatics, Daqing Campus, Harbin Medical University, Daqing 163319, China; The First Affiliated Hospital, Institute of Cardiovascular Disease, Hengyang Medical School, University of South China, Hengyang, Hunan 421001, China; School of Computer, University of South China, Hengyang, Hunan 421001, China; School of Medical Informatics, Daqing Campus, Harbin Medical University, Daqing 163319, China; School of Medical Informatics, Daqing Campus, Harbin Medical University, Daqing 163319, China; The First Affiliated Hospital, Institute of Cardiovascular Disease, Hengyang Medical School, University of South China, Hengyang, Hunan 421001, China; School of Computer, University of South China, Hengyang, Hunan 421001, China; The First Affiliated Hospital, Cardiovascular Lab of Big Data and Imaging Artificial Intelligence, Hengyang Medical School, University of South China, Hengyang, Hunan 421001, China; The First Affiliated Hospital, Institute of Cardiovascular Disease, Hengyang Medical School, University of South China, Hengyang, Hunan 421001, China; School of Medical Informatics, Daqing Campus, Harbin Medical University, Daqing 163319, China; School of Medical Informatics, Daqing Campus, Harbin Medical University, Daqing 163319, China; School of Medical Informatics, Daqing Campus, Harbin Medical University, Daqing 163319, China; School of Medical Informatics, Daqing Campus, Harbin Medical University, Daqing 163319, China; School of Medical Informatics, Daqing Campus, Harbin Medical University, Daqing 163319, China; The First Affiliated Hospital, Institute of Cardiovascular Disease, Hengyang Medical School, University of South China, Hengyang, Hunan 421001, China; School of Medical Informatics, Daqing Campus, Harbin Medical University, Daqing 163319, China; School of Computer, University of South China, Hengyang, Hunan 421001, China; The First Affiliated Hospital, Cardiovascular Lab of Big Data and Imaging Artificial Intelligence, Hengyang Medical School, University of South China, Hengyang, Hunan 421001, China; Hunan Provincial Base for Scientific and Technological Innovation Cooperation, University of South China, Hengyang, Hunan 421001, China; The First Affiliated Hospital, Department of Cardiology, Hengyang Medical School, University of South China, Hengyang, Hunan 421001, China; Department of Biochemistry and Molecular Biology, School of Basic Medical Sciences, Hengyang Medical School, University of South China, Hengyang, Hunan 421001, China; Department of Cell Biology and Genetics, School of Basic Medical Sciences, Hengyang Medical School, University of South China, Hengyang, Hunan 421001, China; The First Affiliated Hospital, Institute of Cardiovascular Disease, Hengyang Medical School, University of South China, Hengyang, Hunan 421001, China; School of Medical Informatics, Daqing Campus, Harbin Medical University, Daqing 163319, China; School of Computer, University of South China, Hengyang, Hunan 421001, China; The First Affiliated Hospital, Cardiovascular Lab of Big Data and Imaging Artificial Intelligence, Hengyang Medical School, University of South China, Hengyang, Hunan 421001, China; Hunan Provincial Base for Scientific and Technological Innovation Cooperation, University of South China, Hengyang, Hunan 421001, China; The First Affiliated Hospital, Department of Cardiology, Hengyang Medical School, University of South China, Hengyang, Hunan 421001, China; Department of Biochemistry and Molecular Biology, School of Basic Medical Sciences, Hengyang Medical School, University of South China, Hengyang, Hunan 421001, China; Department of Cell Biology and Genetics, School of Basic Medical Sciences, Hengyang Medical School, University of South China, Hengyang, Hunan 421001, China

## Abstract

Super-enhancers (SEs) are cell-specific DNA cis-regulatory elements that can supervise the transcriptional regulation processes of downstream genes. SEdb 2.0 (http://www.licpathway.net/sedb) aims to provide a comprehensive SE resource and annotate their potential roles in gene transcriptions. Compared with SEdb 1.0, we have made the following improvements: (i) Newly added the mouse SEs and expanded the scale of human SEs. SEdb 2.0 contained 1 167 518 SEs from 1739 human H3K27ac chromatin immunoprecipitation sequencing (ChIP-seq) samples and 550 226 SEs from 931 mouse H3K27ac ChIP-seq samples, which was five times that of SEdb 1.0. (ii) Newly added transcription factor binding sites (TFBSs) in SEs identified by TF motifs and TF ChIP-seq data. (iii) Added comprehensive (epi)genetic annotations of SEs, including chromatin accessibility regions, methylation sites, chromatin interaction regions and topologically associating domains (TADs). (iv) Newly embedded and updated search and analysis tools, including ‘Search SE by TF-based’, ‘Differential-Overlapping-SE analysis’ and ‘SE-based TF–Gene analysis’. (v) Newly provided quality control (QC) metrics for ChIP-seq processing. In summary, SEdb 2.0 is a comprehensive update of SEdb 1.0, which curates more SEs and annotation information than SEdb 1.0. SEdb 2.0 provides a friendly platform for researchers to more comprehensively clarify the important role of SEs in the biological process.

## INTRODUCTION

Super-enhancers (SEs) are clusters composed of multiple enhancer constituents, which can program and regulate gene expression patterns. Similar to typical enhancers, SEs can recruit transcription factors (TFs), transcriptional co-factors, chromatin regulators and chromatin complexes to participate in transcriptional regulation processes (1–3). The difference is that the length of genome coverage and binding density of the active factors of SEs are several times or even dozens of times that of typical enhancers (4). The transcriptional regulation ability of SEs is much higher than that of typical enhancers (5). Importantly, a large number of studies have revealed that SEs can promote many biological processes, such as tumor occurrence and development, embryonic development, immune response, and cell invasion and metastasis, by regulating key genes in these processes. For example, SEs can promote the development of breast cancer by activating the key gene FOXC1 that enhances the regulation of cancer cell growth and metastasis (6). Moreover, studies have found that DNA methylation, sequence variation, and chromatin accessibility in the SE region could affect the regulatory potential of SEs (7). The dynamic changes of SE activity in different stages of development were related to the changes of chromatin state (8). DNA methylation affects the interaction between protein and DNA by causing changes in DNA conformation, which ultimately inhibits the binding efficiency of TFs and cis-regulatory elements such as SEs, enhancers and promoters (9,10). In megakaryocytes, the common sequence variation in the cell type specific SE region changes platelet function (11). In conclusion, these studies have demonstrated that SEs had great regulatory potential in the development of complex diseases and the maintenance of healthy cell properties and functions.

In the last few years, researchers have developed multiple SE related databases, such as dbSUPER (12) and SEA (13), which provide large amounts of SE information for researchers. For example, dbSUPER contains basic information such as the chromosomal location of the SE regions and related genes from human and mouse. SEA is mainly dedicated to curating multi-species SEs and a small amount of annotation information. We developed SEdb 1.0 in 2019 to annotate with multiple perspectives the functional effects of SEs on gene transcriptional regulation in a cell type-specific manner, which contained a multitude of human SEs and plentiful annotation data of SEs to decipher the transcriptional regulation mechanism (14). Moreover, after the SEdb 1.0 release, the continued rapid accumulation of multi-species ChIP-seq data provided valuable resources for exploring SE functions. In particular, the H3K27ac ChIP-seq data of mouse has accumulated in an unprecedented manner. Mouse models provide important research vehicles both for revealing the pathological molecular mechanisms and for the preclinical evaluation of therapies against a variety of human complex diseases, especially cardiovascular diseases and cancers. Emerging evidence has also demonstrated that mouse SEs play vital regulatory roles in cardiovascular diseases via activating downstream functional genes (15,16). A large number of researchers have discovered that key TFs bind to SEs to supervise the transcriptional regulation capabilities of SEs for cell-specific genes, cancer driver genes and other important genes (17). The complex regulatory relationship between TFs and genes have been believed to be largely mediated by *cis* regulatory elements such as SEs, enhancers and promoters (18,19). Meanwhile, numerous studies have shown that the chromatin features within SE regions, including DNA methylation, chromatin interaction and chromatin accessibility, are synergistic to control gene transcription (20). For instance, integrating ATAC-seq density and SE activity can construct core regulatory circuits and identify core causal TFs in cancers (21). Changes in three-dimensional (3D) chromatin architecture have a strong effect on the integrity of topologically associating domains (TADs) and rewiring specific enhancer–promoter interactions, which can lead to the dysregulated gene expression and cause diseases (22). Researchers revealed that the dynamic methylation of SEs regulated transcriptional heterogeneity (23). Therefore, further integrating these large-scale datasets to explore the functions of SEs and elucidate the transcriptional regulation mechanisms of SEs in different cell/tissue types is urgently required.

**Figure 1.**
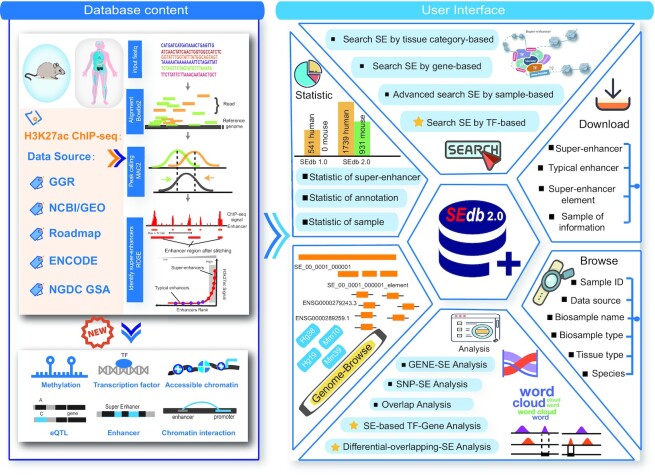
Database content and construction. SEdb2.0 uses multi-source H3K27ac ChIP-seq data to identify human and mouse super-enhancers. New genetic and epigenetic annotations are collected, such as accessible chromatin, methylation sites, chromatin interactions and so on. SEdb 2.0 contains a variety of functions to browse, search, download and visualize super-enhancers.

We developed SEdb 2.0, an updated and significantly expanded database, which introduced the new SEs for mouse and included a major expansion of human SE resources, to provide a more comprehensive collection, interpretation and analysis of SEs. Currently, SEdb 2.0 documented 1 717 744 SEs from 2670 samples, including 541 original human samples, 1198 newly released human samples and 931 mouse samples. Notably, the scales of datasets and SEs have been increased by more than five times compared with SEdb 1.0. Importantly, SEdb 2.0 predicted TF binding within SE regions through TF motif analysis and TF ChIP-seq data. Briefly, we collected and processed TF binding data of >1400 TFs from human and mouse to interpret the SE-related regulatory information across different cell/tissue types. Also, SEdb 2.0 newly extended genetic and epigenetic annotations from multiple perspectives, including chromatin accessibility regions, methylation sites, chromatin interactions regions, and TADs. Furthermore, SEdb 2.0 also added new analysis and search functions, such as ‘Search SE by TF-based’, ‘Differential-Overlapping-SE analysis’ and ‘SE-based TF–Gene analysis’ (Table 1). Collectively, SEdb 2.0 is an easy-to-use platform to curate massive SE data of human and mouse, which provide comprehensive annotations and analysis tools for facilitating the interpretation of SEs (Figure 1).

**Table 1.** SEdb 2.0 data content compared with SEdb 1.0

Function type	Data type/specific function	SEdb 1.0	SEdb 2.0	Fold increase
Interaction table/annotation	Species	Human	Human, Mouse	2
	Sample	542	2670	∼5
	Super-enhancer	331 601	1 717 744	∼5
	Common SNP	38 063 729	79 078 255	∼2
	eQTL	31 080 511	61 786 727	∼2
	Enhancer	14 867 092	79 709 120	∼5
	Chromatin accessibility region	No	Yes	New
	Methylation site	No	Yes	New
	Chromatin interactions region	No	Yes	New
	TAD	No	Yes	New
	TF ChIP-seq	No	Yes	New
	TF motif scan analysis	No	Yes	New
Genome browser	Reference genome	hg19	hg38, hg19 mm10, mm39	4
	Super-enhancer	Yes	Yes	–
	Super-enhancer element	Yes	Yes	–
	TFBS by ChIP-seq	No	Yes	New
	Methylation site	No	Yes	New
	TAD	No	Yes	New
	SNP	Yes	Yes	–
	Common SNP	Yes	Yes	–
	Risk SNP	Yes	Yes	–
	TFBS conserved	Yes	Yes	–
	CRISPR/Cas9 target site	Yes	Yes	–
	DHS	Yes	Yes	–
	Enhancer	Yes	Yes	–
	Conservative score	Yes	Yes	–
Analysis functions	Differential-Overlapping-SE analysis	No	Yes	New
	SE-based TF–Gene analysis	No	Yes	New
	Gene-SE analysis	Yes	Yes	–
	SNP-SE analysis	Yes	Yes	–
	Overlap analysis	Yes	Yes	–
	Region analysis^a^	Yes	Yes	–

^a^External link to GREAT.

## DATA EXPANSION AND PRE-PROCESSING

### Data collection and identification of SEs

The main improvements from SEdb 1.0 to SEdb 2.0 in this section are the addition of mouse SE sets, the extension of human SE scale, and optimized SE identification workflow. SEdb 2.0 included 1739 human H3K27ac ChIP-seq samples and 931 mouse H3K27ac ChIP-seq samples. We processed the newly collected data with updated workflow and reference genomes. Briefly, we firstly collected all H3K27ac ChIP-seq and corresponding input control sequencing data from NCBI GEO/SRA (24,25), ENCODE (26), Roadmap (26,27), Genomics of Gene Regulation Project (GGR) (26) and National Genomics Data Center Genome Sequence Archive (NGDC GSA) (28,29). It is worth mentioning that GSA is a new data source added in SEdb 2.0 and a new data repository for raw sequence reads in China. Second, for genome alignment, we replaced the previously used Bowtie (v0.12.9) (30) with Bowtie 2 (v2.4.4), which is more efficient and more suitable for ChIP-seq data. Meanwhile, the reference genome in alignment has also been upgraded (Human hg38 and Mouse mm10). Finally, for peak calling, we replaced MACS14 (31) with the optimized MACS2 (2.2.7) (Supplementary Table S1). We identified 1,167,518 human SEs and 550 226 mouse SEs with the upgraded workflow. Furthermore, for the SEs contained in SEdb1.0, we converted all the SE regions into the hg38 genome using the liftOver (http://genome.ucsc.edu/cgi-bin/hgLiftOver) tool of UCSC (32). In addition, since the ChIP-seq data come from different experiments using different antibodies, the quality of the ChIP-seq data becomes uneven. We used ChIPQC to calculate some quality assessment metrics, such as the SSD score, cross-coverage score at the fragment length, and percentage of reads within peaks, to generate a quality report for each ChIP-seq data. The generated quality report was displayed in the sample details page for user's reference.

### Identification of TF binding sites on SEs

TF binding is an important ability for SEs to participate in gene transcription. We used two strategies to further investigate the relationship between SEs and TFs in the transcriptional regulation process. One was TF ChIP-seq data based prediction, and the other was TF motif scan analysis. For TF ChIP-seq data based prediction, we collected 51 616 973 non-redundant binding regions from 817 human TFs and 32 985 444 non-redundant binding regions of 648 mouse TFs across various cell lines and tissue types from ReMap 2022 (33). Next, due to the special structure of SEs, we separated the SE element regions to more precisely find TF binding sites that bound to SEs. Finally, we used BEDTools (34) to perform the region overlap analysis for SE element regions and peaks from TF ChIP-seq data to identify potentially bound TFs. For TF motif scan analysis, we first collected the position weight matrices of TF motif from multiple sources, including Jolma2013 (35), JASPAR CORE 2020 vertebrates (36), Homeodomains (37), UniPROBE (38), and Wei2010 (39). Second, Find Individual Motif Occurrences (FIMO) (40), which is the part of the MEME (41) Suite software toolkit, was used to scan for the occurrences of motifs within every SE element region. Finally, we identified potential TFs binding to SEs with a *P*-value threshold of 1.0E–06.

### Genetic and epigenetic annotations of SEs

Genetic and epigenetic annotations within the SE regions affect the transcriptional regulatory ability of SE. We extended the annotation data of SEs in both quantity and type. Besides the abundant annotation information including common single-nucleotide polymorphisms (SNPs), motif changes, eQTLs, risk SNPs, TFBSs, CRISPR/Cas9 target sites, DNase I hypersensitive signals (DHSs) and enhancers provided by SEdb 1.0, SEdb 2.0 added more comprehensive genetic and epigenetic annotation information, including chromatin accessibility regions, methylation sites, chromatin interaction regions and TADs. Moreover, we also updated and extended the original annotation information in SEdb 1.0, such as common SNPs, eQTL-gene pairs and enhancers.

#### Chromatin accessibility regions

It was found that the complex relationships between the SEs and chromatin accessibility regions would help analyze the combination of TFs and the gene expression mechanism. Therefore, SEdb 2.0 added >130 000 000 chromatin accessibility regions from ATACdb (42), which was the previous job of our research group. Briefly, we manually collected 2723 samples to cover multiple tissues or cell types from NCBI GEO/SRA (24,25) and used Bowtie2 and MACS2 to identify chromatin accessibility regions.

#### Chromatin interactions/TADs

The complex three-dimensional chromatin landscapes formed by chromatin interaction are considered highly credible evidence for SEs to regulate downstream gene expression. We obtained 34 342 926 human chromatin interactions from Oncobase (43) and 4DGenome (44) and obtained 93 516 mouse chromatin interaction data from 4DGenome (44). TADs are also evidence of chromatin interaction, which can directly reveal the relationship between SEs and gene promoters (45,46). A total of 72 019 human TADs covering 21 tissues or cell lines were obtained from the 3D Genome Browser (47).

#### Methylation

DNA methylation status on gene cis-regulatory elements changes the binding density of TFs and further determines the transcriptional activity of genes. Therefore, annotating methylation sites in SE regions is extremely important. SEdb 2.0 collected a large amount of methylation data from two different technologies, which were 450K array and whole-genome shotgun bisulfite sequencing (WGBS). We downloaded 32 099 124 human methylation sites from 450k arrays and 176 535 822 human WGBS datasets from ENCODE (26).

#### Common SNPs/eQTLs/SNPs

SNPs in SE regions can affect the ability of SEs to regulate genes. Due to the upgrade of common SNP and eQTL related databases, we also re-collected the mutation data to provide up-to-date annotations. For example, the number of common SNPs (38063 729 to 79 078 255) obtained from dbSNP release 151 was about twice that of the previous ones (48). Also, eQTL-gene pairs were increased from 31 080 511 to 61 786 727. Besides, we collected 81,432,271 mouse SNPs from dbSNP release 151.

#### Enhancers

SEs are constituted by multiple active enhancers. We also expanded the source of enhancer to achieve a better understanding of the regulatory roles of SEs. The enhancer data sets from EnhancerAtlas (49), FANTOM5 (50), ENCODE (26), HANCER (51), DENdb (52) and ENdb (53) were collected and processed in this step, which contained 79,664,341 human enhancers and 44,779 mouse enhancers. Of these, a majority of enhancer sets were identified using high-throughput experimental data, such as FANTOM5 (50) and ENCODE (26). Notably, a subset of experimentally validated enhancers ENdb (53) were also collected, which were manually curated based on low-throughput experimental data.

## DATABASE IMPROVED USER INTERFACE

### Search function of novel perspective

SEdb 2.0 is a data platform with a more friendly search function. Four query methods exist that can inquire about SEs, including ‘Search SE by Tissue-Category-based’, ‘Search SE by gene-based’, ‘Search SE by genomic region’ and ‘Search SE by TF-based’. Among these, the TF-based query was newly added in SEdb 2.0 (Supplementary Figure S1B). The users can determine the scope of the SE query by selecting species and strategies and inputting TF names. In the result page, the users can retrieve the summary information of TF-based SE search results. TF overview, expression and disease details are also displayed. In the table of SE results, SEdb 2.0 provides the TF name, number of SEs bound by TFs in each sample, and detailed sample information, including sample ID, biosample type, tissue type, and biosample name. The users can click ‘detail’ to view the detailed information of TF associated SEs in the current sample, such as the SE region and SE ID. We also optimized the result pages of the previous three query results. For example, we added SE annotation visualization and the wordcloud of SE associated gene/TFs on the detail page of the SE sample. Meanwhile, we also added an SE associated network and candidate TF binding regions within SE regions predicted by motif analysis and TF ChIP-seq data on the detail page of the SE (Supplementary Figure S1A).

### New ‘Analysis’ interface for Differential-Overlapping-SE analysis tool

SEdb1.0 provided three useful analysis tools to help investigate the SE functions in multiple perspectives, including ‘Gene-SE analysis’, ‘SNP-SE analysis’ and ‘Overlap analysis’. Importantly, SEs are considered as cell-specific DNA regulatory elements. We thus added a fourth analysis function named as ‘Differential-Overlapping-SE analysis’ to explore the differences between SEs from different cell types and disease states. In the ‘Differential-Overlapping-SE analysis’ function, the users can select two samples of interest from the same species based on the hierarchy between tissue types and samples. Simultaneously, SEdb 2.0 uses BEDTools (34) to compare reference genome locations to online analyze differential and overlapped SEs between the two submitted samples. In the result page, SEdb 2.0 can display detailed SE information, such as differential and overlapped SE regions, region length, overlapping ratio and SE associated genes. At the same time, two buttons (Gene Ontology (54) enrichment and KEGG (55) pathway enrichment) are provided for each sample to realize online enrichment analysis of specific SE associated genes using clusterProfiler (56) package in the current sample (Supplementary Figure S1C).

### New ‘Analysis’ interface for SE-based TF–Gene analysis tool

Deciphering the complex relationship among TFs, SEs and genes in transcriptional regulatory mechanisms is the key to understanding the occurrence and development of diseases and biological processes. We provided the fifth analysis tool called SE-based TF–Gene analysis to better discover the relationship among them. When the users submit two lists of TF and gene and select species of interest, SEdb 2.0 identifies SE-mediated TF–gene pair(s) in different tissue types. Meanwhile, the users can set different statistical thresholds through the ‘FIMO’ option. On the analysis result page, SEdb 2.0 provides a drop-down box for selecting tissue type to view results from different tissue types. Numbers, genomic regions, and sample information of TF–gene pair(s) associated SEs can be displayed (Supplementary Figure S1D).

### Upgrade genome browser

We also made a comprehensive upgrade of the original genome browser. Current genome browser contained multiple reference genomes per species, including human (hg38 and hg19) and mouse (mm10 and mm39). Respectively, hg38 and mm10 are displayed as default in human and mouse. At the same time, many new tracks such as TFBS by ChIP-seq data and TADs are embedded.

### Diverse data download

SEdb 2.0 upgraded the reference genomes and provided multiple reference genomes, including hg19, hg38, mm10 and mm39 of SEs, typical enhancers and SE elements of all samples for download on the ‘Download’ page. It is worth noting that SEdb 2.0 also provided the package download of SEs, SE associated genes/TFs, SE elements, TEs based on different tissue/cell types. Moreover, the genetic and epigenetic annotations are also provided for download, such as common SNPs, risk SNPs, eQTLs, TADs, and DHSs. In addition, all the results obtained in the SE details page, search results page, and analysis results page can be downloaded through the ‘Download’ button.

### Case study

#### Case study of SE-based TF–Gene analysis

**Figure 2.**
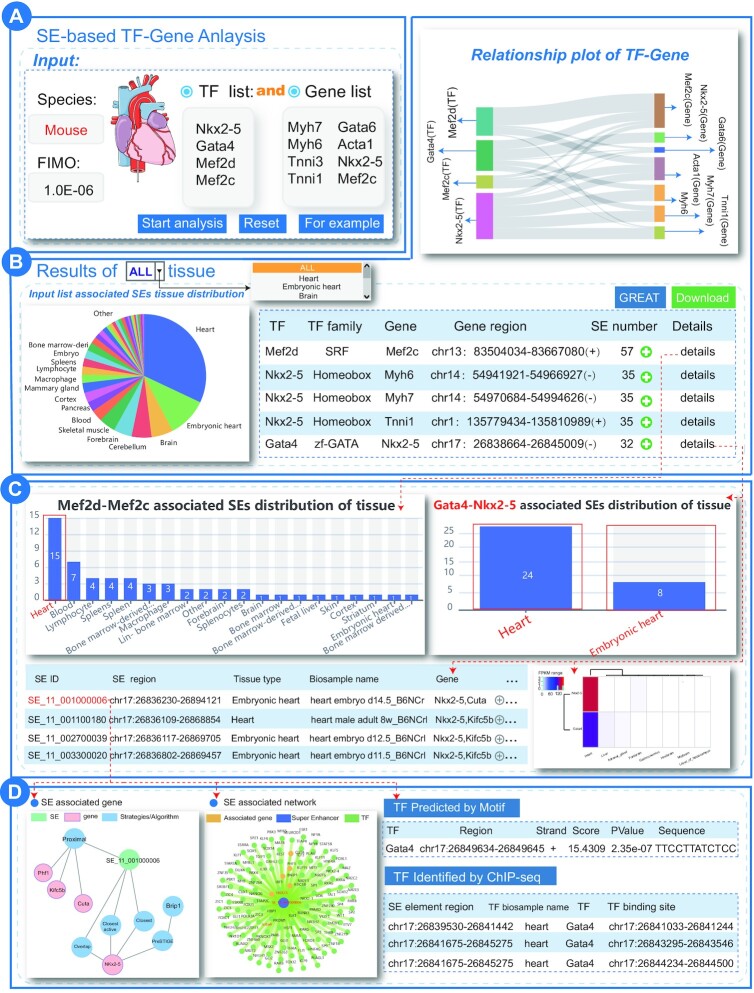
Results of ‘SE-based TF–Gene analysis’ for mouse cardiac enriched TF list and marker gene list. (**A**) User submits gene list and TF list of interest. (**B**) Results overview for SE-based TF–Gene Analysis. (**C**) TF–gene pair associated super-enhancer details. (**D**) Detailed results for super-enhancer ‘SE_11_001000006’.

We performed ‘SE-based TF–Gene analysis’ by inputting mouse cardiac enriched TFs (Nkx2-5, Gata4, Mef2c and Mef2d) as TF input and mouse cardiac marker genes (Myh7, Myh6, Tnni3, Tnni1, Gata6, Acta1, Nkx2-5 and Mef2c) as gene input list to exhibit the new usage and potential applications of SEdb 2.0 in mouse model-based research fields, especially in cardiovascular diseases. We set the analysis parameters as species: mouse and FIMO: 1.0E–06 (Figure 2A). The analysis results showed a complex regulatory relationship between these TFs and the genes mediated by SEs (Figure 2B). The tissue distribution map of these SEs related to TF–gene pairs showed that most of them were distributed in heart-related tissues. For example, SEs enriched by Mef2d-Mef2c, Nkx2-5-Myh6, Myh7 and Gata4-Mef2c pairs were mainly distributed in the heart tissue. Intriguingly, Gata4-Nkx2-5, Myh6 and Myh7 pairs were distributed only in the heart or embryonic heart tissue (Figure 2C). This was consistent with previous findings of SEs in mediating TF–gene regulatory mechanisms, demonstrating that the Nkx2-5 upstream enhancer had a high-affinity binding site for the TF Gata4, thus activating and enhancing the transcription of Nkx2-5 in cardiac development (57,58). Meanwhile, normal Gata4 and Nkx2-5 activities might drive normal cardiac development and the mutation events on Gata4 and Nkx2-5 were considered as the causes of the occurrence and development of congenital heart disease (59,60). By clicking details, the users can further obtain the distribution of SEs that potentially mediate the current TF–gene pairs in various tissues and their general information. If the users want to view more detailed information about SEs, including SE overview, SE annotation and other genes and TFs associated with SEs, they can click SE ID, such as ‘SE_11_001000006’, ‘SE_11_001100180’ and so on. For SE ‘SE_11_001000006’ the detailed result showed that multiple GATA4-binding sites were identified in this SE region by overlapping cardiac related GATA4 TF ChIP-seq data, which further confirmed the regulatory relationship between GATA4, Nkx2-5 SEs (SE_11_001000006) and Nkx2-5 (Figure 2D). Most of the transcriptional regulatory axes composed of TFs and genes have been demonstrated to be regulated by SEs or enhancers. The dysregulation of these pairs can affect heart development and the occurrence and development of cardiovascular diseases. In general, SEdb 2.0 can help users further understand the regulatory relationship between TFs and genes mediated by SEs.

#### Case study of Differential-Overlapping-SE analysis

**Figure 3.**
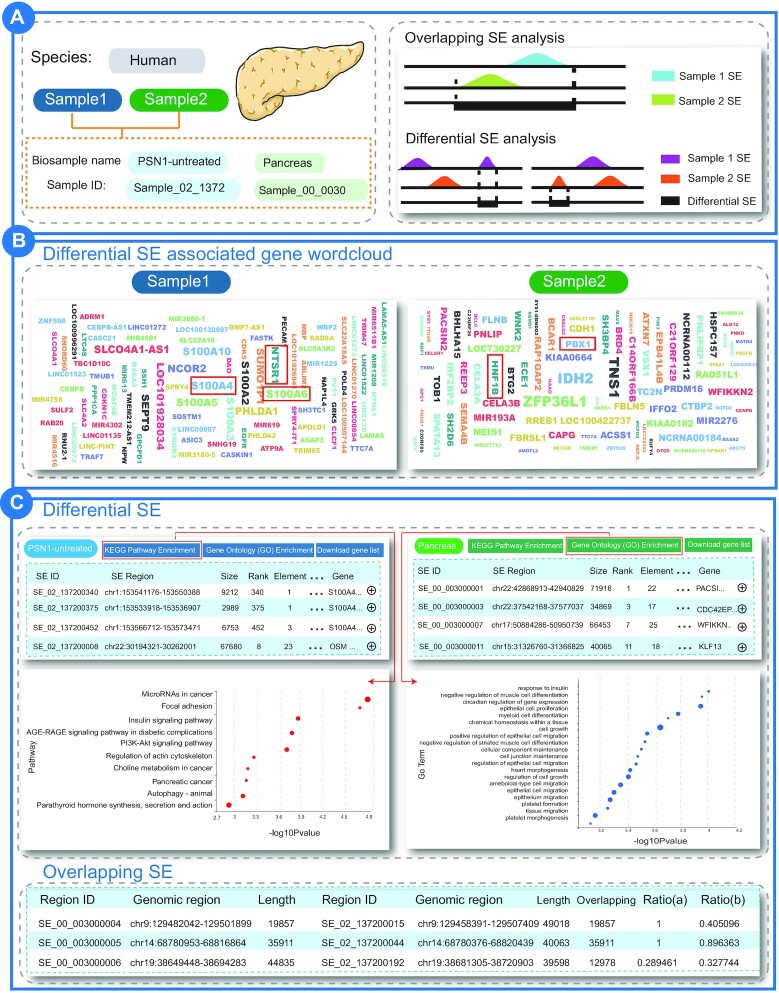
Results of ‘Differential-Overlapping-SE analysis’ between PSN1 and healthy pancreas tissues. (**A**) User selects two samples of interest. (**B**) Wordcloud map of specific super-enhancer associated genes in samples. (**C**) Differential super-enhancers and overlapping super-enhancers.

We selected two human samples, including pancreatic cancer cell line PSN1 (Biosample_name: PSN1-untreated, Sample ID: Sample_02_1372) and healthy pancreas tissues (Biosample_name: pancreas, Sample ID: Sample_00_0030) as input to highlight the usage of ‘Differential-Overlapping-SE analysis’ (Figure 3A). A total of 469 specific SEs in pancreatic cancer cell line PSN1 and 383 specific SEs in healthy pancreas tissues were identified in this analysis. As expected, many differential SE-associated genes in PSN1 were the key genes of pancreatic cancer, such as S100A4, S100A6, S100A2, NTSR1 and CDK5 (Figure 3B). Studies showed that S100 calcium binding protein A (S100A) family members were associated with the occurrence and development of pancreatic cancer (61). Among these, S100A4 promoted pancreatic cancer progression and accelerated cell motility by activating the Src-FAK-mediated dual signaling pathway in pancreatic cancer cells (62,63). Moreover, the inhibition of S100A6 expression reduced the proliferation and invasiveness of pancreatic cancer cells (64). Thus, S100 calcium-binding protein A family members are increasingly recognized as diagnostic markers and therapeutic targets for pancreatic cancer (65–68). Meanwhile, NTSR1 is also considered to be closely related to pancreatic cancer. The overexpression of NTSR1 induced high tumorigenic and metastatic capacity in pancreatic cancer cells (69). We obtained the pathway results annotated by differential SE-associated genes in the PSN1 cell line by clicking the KEGG pathway enrichment button of sample PSN1-untreated. The pathways significantly enriched by these specific SE associated genes in pancreatic cancer are microRNAs in cancer, mocal adhesion, insulin signaling pathway, PI3K-Akt signaling pathway, pancreatic cancer, regulation of actin cytoskeleton, MAPK signaling pathway and other cancer related pathways (Figure 3C). PI3K-Akt signaling pathway is abnormally activated in pancreatic cancer, affecting multiple biological processes such as cancer cell cycle progression and cellular metabolic rate, and associated with poor prognosis of patients (69–71). Indeed, Li *et al.* demonstrated that scoparone targeted the PI3K/Akt signaling pathway to induce cell cycle arrest and apoptosis in pancreatic cancer cells (72). However, the specific SE associated genes in healthy pancreas tissues are generally associated with pancreatic development, such as HNF1B, IPF1 and PBX1 (73) (Figure 3B). For example, HNF1B can control pancreatic pluripotent progenitor cell expansion, as well as pancreatic morphogenesis in mouse (74). HNF1B heterozygous mutations were associated with pancreatic hypoplasia in human (75). Furthermore, the Gene Ontology enrichment analysis of these specific SE associated genes in healthy pancreatic tissues showed that these SE associated genes were annotated in development related biological processes, such as cell growth, epithelial cell proliferation and migration. In conclusion, these analyses in our database could identify specific SE target genes associated with different phenotypes, showing that SEdb 2.0 is valuable in exploring the differences between different tissues, cell types, and phenotypes from the perspective of SEs.

In addition, we also performed ‘Search super-enhancers by TF-based’ to search mouse TF Nanog. The results showed that the SEs bound by TF Nanog were enriched in mouse embryonic stem cell samples, and these SEs and their related genes constituted the specific core pluripotent regulatory network to embryonic stem cells (Supplementary Materials and Supplementary Figure S2). These findings suggested that SEdb 2.0 was useful in finding SEs of interest by searching TF.

## CONCLUSIONS AND FUTURE EXTENSIONS

In recent years, the key role of SEs in cell identity, disease development, and biological processes has been found and well investigated via low-throughput experiments (6,76). We developed the update of SEdb 2.0 by processing a large number of H3K27ac ChIP-seq data from new tissues and cell types to expand the human SE scale and introduce the SEs of mouse to provide more regulatory cues for human and mouse SEs. We also identified TF binding sites within SE regions via two strategies: TF motif scan analysis and TF ChIP-seq data. At the same time, SEdb 2.0 extended more comprehensive genetic and epigenetic annotations within SE regions and provided more useful query and analysis functions to interpret SE mediated transcriptional regulation. Among these, the GREAT (77) based genomic region annotation function of search results connects the position of SE on the genome with its biological function. Differential-Overlapping-SE analysis tool can help researchers find disease specific SE regions and provide potential targets for disease treatment and prognosis. Also, SE-based TF–Gene analysis tool interprets the complex interaction between TFs and genes from the perspective of SEs. The newly added genetic and epigenetic annotations of SEs, such as chromatin accessible regions, DNA methylation and so on, provide a multi-dimensional and in-depth perspective to understand the regulatory mechanism of SEs. In general, the updated SEdb enables the users to better understand the key role of SEs in the occurrence of complex diseases, embryonic development, immune response and other biological processes.

In the future, we will continue to update and maintain SEdb. SEdb will be improved in the following three directions. First, SEdb will collect more H3K27ac ChIP-seq data from different tissue/cell types and disease phenotypes and further increase SE-related annotation information. Second, as the relationship between SEs and diseases is gradually revealed, SE-targeted therapy has become the novel clinical treatment options, especially in multiple cancers. We will follow up the investigations between drugs and SEs and integrate these data in the future version of SEdb. Finally, single-cell sequencing technology, such as scATAC-seq and scCUT&Tag (78), continues to develop and enabling the identification of cell-specific SEs at single cell resolution. We will continue to track the development of this field to achieve large-scale single-cell level SEs recognition and curation. We believe that SEdb will facilitate SE research with more researchers.

## DATA AVAILABILITY

The research community can access information freely in the SEdb 2.0 without registration or logging in. The URL for SEdb 2.0 is http://www.licpathway.net/sedb/.

## Supplementary Material

gkac968_Supplemental_Files
