# Supplementary material for: SEdb 2.0: a comprehensive super-enhancer database of human and mouse
Source: Nucleic Acids Res. 2022 Nov 1;51(D1):D280–90. doi: 10.1093/nar/gkac968 (PMC9825585; doi:10.1093/nar/gkac968)
Supplement: gkac968_Supplemental_Files [file gkac968_supplemental_files.zip › Revised_Supplementary Materials.docx]

**Supplementary material of SEdb 2.0: a comprehensive super-enhancer database of human and mouse**

***Case study of Search super-enhancers by TF-based*** We used mouse TF Nanog as the input of ‘Search super-enhancers by TF-based’ to illustrate how to use the newly added search function of SEdb 2.0, and selected two strategies including ‘TF ChIP-seq’ and ‘Motif scan’ (Strategies/Algorithm: ALL) to query. On the result page, SEdb 2.0 first provided basic information related to TF, such as TF family, TF name, ensembl ID, entrez ID, and so on. Second, the search results of “TF ChIP-seq” strategy to find SEs showed that the binding sites of Nanog in the SE regions from mouse embryonic stem cells were the maximum, such as sample ‘Sample_12_0258: ESCs WT’, ‘Sample_12_0128: E14Tg2a treat 6h’, ‘Sample_12_0259: ESCs Dnttip1 KO2’, and ‘Sample_12_0297:E14 Pml-/-’, and most of these TFBSs were derived from TF ChIP-seq data of mouse embryonic stem cells. This was consistent with previous studies that TF Nanog controlled the self-renewal and pluripotency of embryonic stem cells by binding to SEs and enhancers to control the expression pattern of pluripotent genes ([1](#_ENREF_1),[2](#_ENREF_2)). We further clicked the plus sign to view the SE information found under the current sample and selected the SE of interest, for instance SE ‘SE_12_025800003’, to enter the SE details page. On the ‘SE_12_025800003’ details page, the Super-enhancer associated network provided by SEdb 2.0 showed that the genes associated with the SE bound by Nanog included Tgif1, Dlgap1 and Myl12b. Studies have shown that the specific enhancer of mouse embryonic stem cells interacts with the promoter region of Tgif1. The expression level of Tgif1 is regulated by the core pluripotency factors in mouse embryonic stem cells, such as Nanog, Sox2, and Oct4, which together constitute the core pluripotent regulatory network in mouse embryonic stem cells. The balance of this network regulation relationship is essential to maintain normal ESC phenotype and induced pluripotency ([3-5](#_ENREF_3)). The ‘Motif scan’ based SEs were massively enriched in embryonic stem cell samples. Finally, SEdb2.0 also provided TF expression in different tissues and cell lines, as well as TF related disease information. It is worth noting that Nanog is highly expressed in embryonic stem cells and is associated with embryos and embryonic tumors. In general, above results confirmed the usefulness of the newly added search function.

**REFERENCES**

1. Whyte, W.A., Orlando, D.A., Hnisz, D., Abraham, B.J., Lin, C.Y., Kagey, M.H., Rahl, P.B., Lee, T.I. and Young, R.A. (2013) Master transcription factors and mediator establish super-enhancers at key cell identity genes. *Cell*, **153**, 307-319.

2. Tsai, P.H., Chien, Y., Wang, M.L., Hsu, C.H., Laurent, B., Chou, S.J., Chang, W.C., Chien, C.S., Li, H.Y., Lee, H.C. *et al.* (2019) Ash2l interacts with Oct4-stemness circuitry to promote super-enhancer-driven pluripotency network. *Nucleic Acids Res*, **47**, 10115-10133.

3. Kim, J., Chu, J., Shen, X., Wang, J. and Orkin, S.H. (2008) An extended transcriptional network for pluripotency of embryonic stem cells. *Cell*, **132**, 1049-1061.

4. Lee, B.K., Shen, W., Lee, J., Rhee, C., Chung, H., Kim, K.Y., Park, I.H. and Kim, J. (2015) Tgif1 Counterbalances the Activity of Core Pluripotency Factors in Mouse Embryonic Stem Cells. *Cell Rep*, **13**, 52-60.

5. Kieffer-Kwon, K.R., Tang, Z., Mathe, E., Qian, J., Sung, M.H., Li, G., Resch, W., Baek, S., Pruett, N., Grontved, L. *et al.* (2013) Interactome maps of mouse gene regulatory domains reveal basic principles of transcriptional regulation. *Cell*, **155**, 1507-1520.

**Supplementary Materials.** Case study of Search super-enhancers by TF-based.

**Supplementary Table S1.** Software and parameters used by SEdb 2.0 to identify super-enhancers.

**Supplementary Figure S1.** New features added in SEdb2.0. (A) New feature on super-enhancers details page including super-enhancers associated network, super-enhancer annotation and TF Binding to super-enhancers. (B) Search super-enhancer by TF-based. (C) Differential-overlapping-SE Analysis. (D) SE-based TF-Gene Analysis.

**Supplementary Figure S2.** Results of search super-enhancers by mouse TF Nanog. (A) Input list for ‘Search super-enhancers by TF-based’. (B) Nanog overview. (C) Super-enhancer search result of Nanog by ‘TF ChIP-seq’ and ‘Motif scan’. (D) Super-enhancer associated network, super-enhancer associated genes and result of overlap with other super-enhancers about SE_ 12_ 025800003. (E) Expression and disease information of Nanog.
