# Supplementary material for: SEdb 2.0: a comprehensive super-enhancer database of human and mouse
Source: Nucleic Acids Res. 2022 Nov 1;51(D1):D280–90. doi: 10.1093/nar/gkac968 (PMC9825585; doi:10.1093/nar/gkac968)
Supplement: gkac968_Supplemental_Files [file gkac968_supplemental_files.zip › Supplementary Figure S1.pdf]

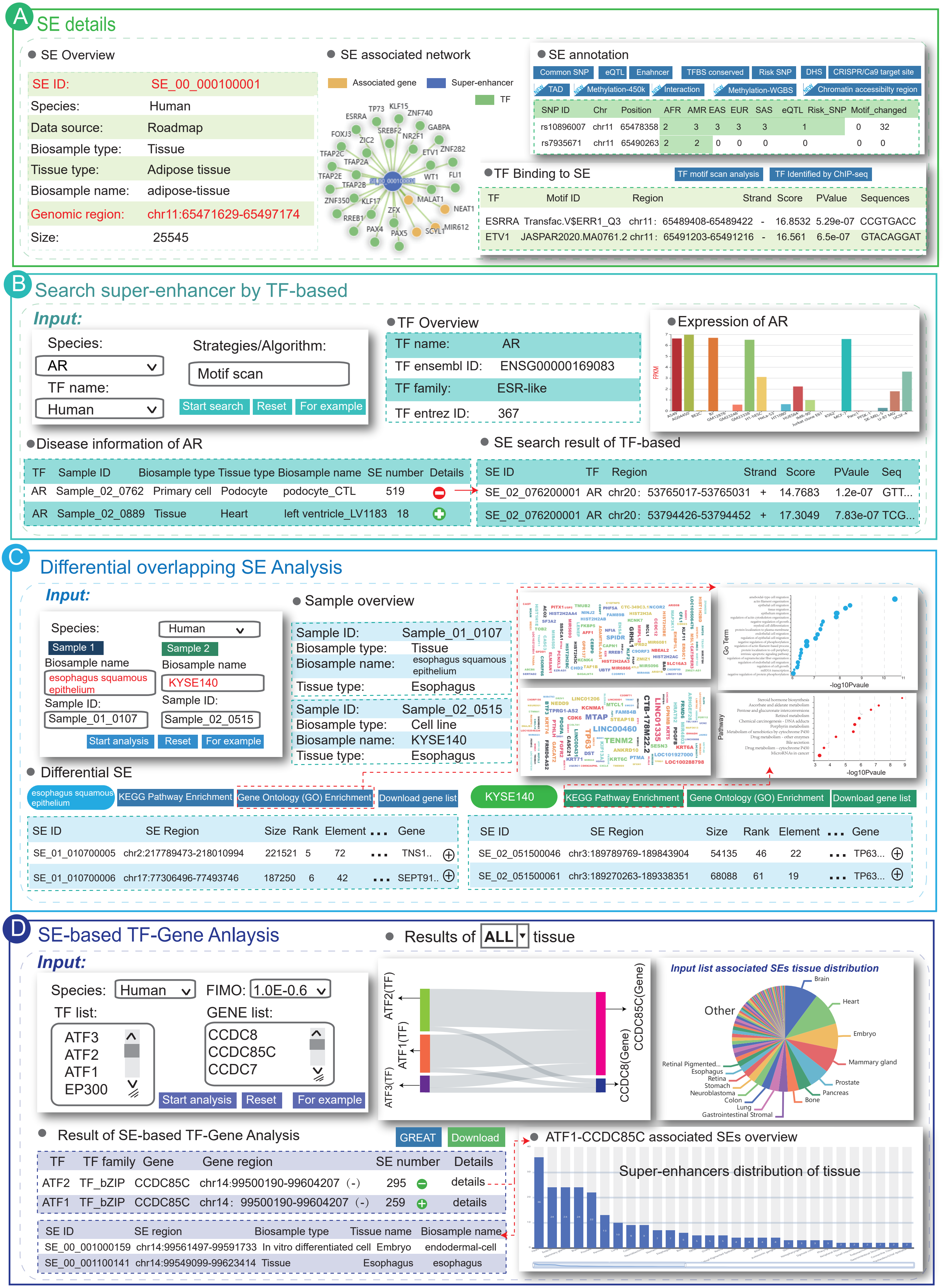

A
SE details
SE Overview
SE ID: SE_00_000100001
Species: Human
Data source: Roadmap
Biosample type: Tissue
Tissue type: Adipose tissue
Biosample name: adipose-tissue
Genomic region: chr11:65471629-65497174
Size: 25545
SE associated network
Associated gene
Super-enhancer
TF
SE annotation
Common SNP
eQTL
Enahncer
TFBS conserved
Risk SNP
DHS
CRISPR/Ca9 target site
TAD
Methylation-450k
Interaction
Methylation-WGBS
Chromatin accessibilty region
SNP ID Chr Position AFR AMR EAS EUR SAS eQTL Risk_SNP Motif_changed
rs10896007 chr11 65478358 2 3 3 3 3 1 0 32
rs7935671 chr11 65490263 2 2 0 0 0 0 0 0
TF Binding to SE
TF motif scan analysis
TF Identified by ChIP-seq
TF Motif ID Region Strand Score PValue Sequences
ESRRA Transfac.V$ERR1_Q3 chr11: 65489408-65489422 - 16.8532 5.29e-07 CCGTGACC
ETV1 JASPAR2020.MA0761.2 chr11: 65491203-65491216 - 16.561 6.5e-07 GTACAGGAT
B
Search super-enhancer by TF-based
Input:
Species:
AR
TF name:
Human
Strategies/Algorithm:
Motif scan
Start search
Reset
For example
TF Overview
TF name: AR
TF ensembl ID: ENSG00000169083
TF family: ESR-like
TF entrez ID: 367
Expression of AR
FPKM
Disease information of AR
TF Sample ID Biosample type Tissue type Biosample name SE number Details
AR Sample_02_0762 Primary cell Podocyte podocyte_CTL 519
AR Sample_02_0889 Tissue Heart left ventricle_LV1183 18
SE search result of TF-based
SE ID TF Region Strand Score PValue Seq
SE_02_076200001 AR chr20: 53765017-53765031 + 14.7683 1.2e-07 GTT...
SE_02_076200001 AR chr20: 53794426-53794452 + 17.3049 7.83e-07 TCG...
C
Differential overlapping SE Analysis
Input:
Species:
Human
Sample 1
Sample 2
Biosample name
esophagus squamous epithelium
KYSE140
Sample ID:
Sample_01_0107
Sample_02_0515
Start analysis
Reset
For example
Sample overview
Sample ID: Sample_01_0107
Biosample type: Tissue
Biosample name: esophagus squamous epithelium
Tissue type: Esophagus
Sample ID: Sample_02_0515
Biosample type: Cell line
Biosample name: KYSE140
Tissue type: Esophagus
Go Term
-log10Pvaule
Pathway
-log10Pvaule
Differential SE
esophagus squamous epithelium
KEGG Pathway Enrichment
Gene Ontology (GO) Enrichment
Download gene list
KYSE140
SE ID SE Region Size Rank Element ... Gene
SE_01_010700005 chr2:217789473-218010994 221521 5 72 ... TNS1..
SE_01_010700006 chr17:77306496-77493746 187250 6 42 ... SEPT91..
SE_02_051500046 chr3:189789769-189843904 54135 46 22 ... TP63...
SE_02_051500061 chr3:189270263-189338351 68088 61 19 ... TP63...
D
SE-based TF-Gene Anlaysis
Input:
Species: Human
FIMO: 1.0E-0.6
TF list:
ATF3
ATF2
ATF1
EP300
GENE list:
CCDC8
CCDC85C
CCDC7
Start analysis
Reset
For example
Results of ALL tissue
ATF2(TF)
ATF1(TF)
ATF3(TF)
CCDC85C(Gene)
CCDC8(Gene)
Input list associated SEs tissue distribution
Brain
Heart
Embryo
Mammary gland
Prostate
Pancreas
Bone
Other
Retinal Pigmented...
Esophagus
Retina
Stomach
Neuroblastoma
Colon
Lung
Gastrointestinal Stromal
Result of SE-based TF-Gene Analysis
GREAT
Download
TF TF family Gene Gene region SE number Details
ATF2 TF_bZIP CCDC85C chr14:99500190-99604207 (-) 295 details
ATF1 TF_bZIP CCDC85C chr14: 99500190-99604207 (-) 259 details
SE ID SE region Biosample type Tissue name Biosample name
SE_00_001000159 chr14:99561497-99591733 In vitro differentiated cell Embryo endodermal-cell
SE_00_001100141 chr14:99549099-99623414 Tissue Esophagus esophagus
ATF1-CCDC85C associated SEs overview
Super-enhancers distribution of tissue
