# Supplementary material for: SEdb 2.0: a comprehensive super-enhancer database of human and mouse
Source: Nucleic Acids Res. 2022 Nov 1;51(D1):D280–90. doi: 10.1093/nar/gkac968 (PMC9825585; doi:10.1093/nar/gkac968)
Supplement: gkac968_Supplemental_Files [file gkac968_supplemental_files.zip › Supplementary Figure S2.pdf]

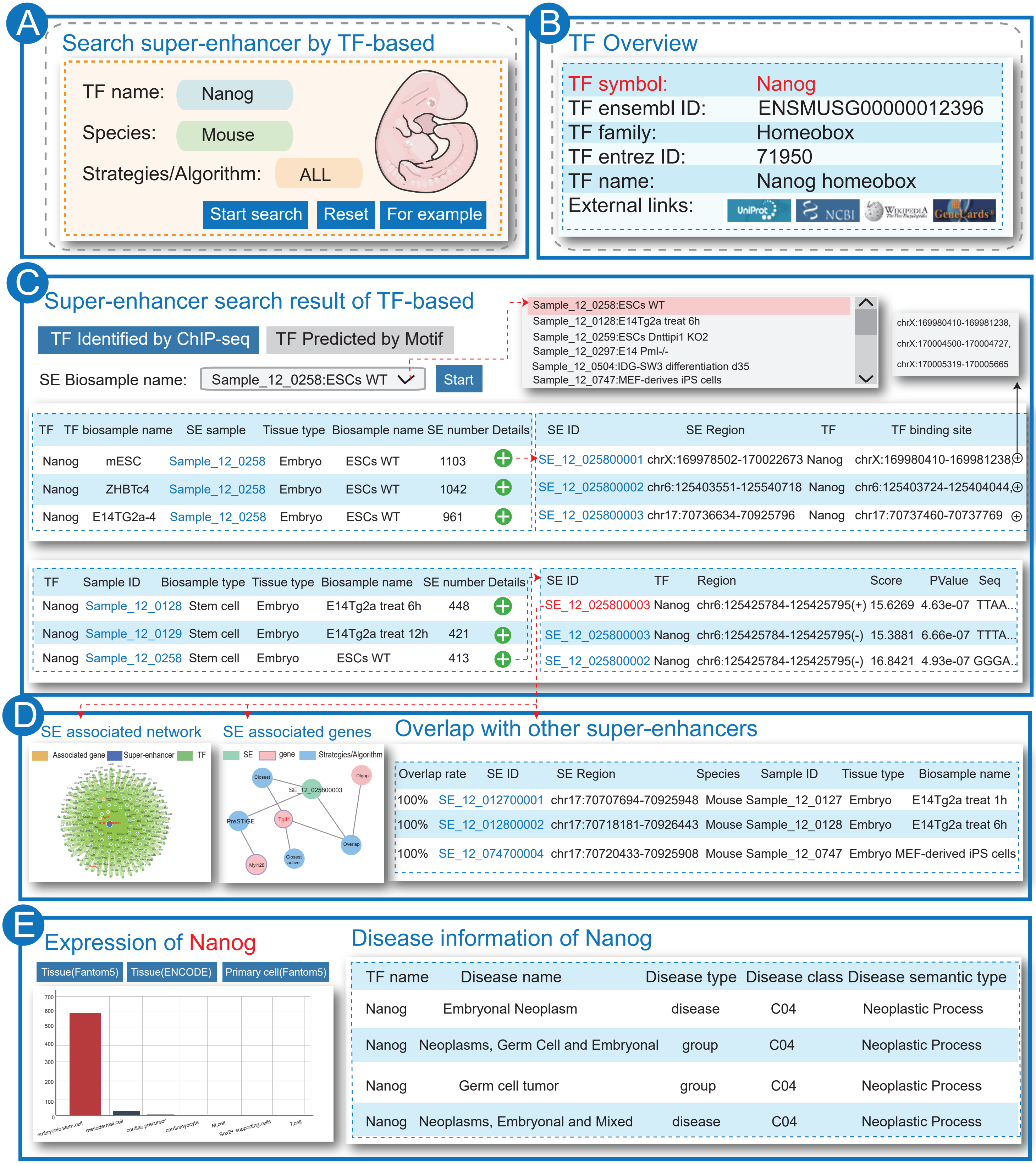

A
Search super-enhancer by TF-based
TF name: Nanog
Species: Mouse
Strategies/Algorithm: ALL
Start search
Reset
For example
B
TF Overview
TF symbol: Nanog
TF ensembl ID: ENSMUSG00000012396
TF family: Homeobox
TF entrez ID: 71950
TF name: Nanog homeobox
External links:
UniProt
NCBI
Wikipedia
GeneCards
C
Super-enhancer search result of TF-based
TF Identified by ChIP-seq
TF Predicted by Motif
SE Biosample name: Sample_12_0258:ESCs WT
Start
Sample_12_0258:ESCs WT
Sample_12_0128:E14Tg2a treat 6h
Sample_12_0259:ESCs Dnttipi1 KO2
Sample_12_0297:E14 Pml-/-
Sample_12_0504:IDG-SW3 differentiation d35
Sample_12_0747:MEF-derives iPS cells
chrX:169980410-169981238,
chrX:170004500-170004727,
chrX:170005319-170005665
TF | TF biosample name | SE sample | Tissue type | Biosample name | SE number | Details
Nanog | mESC | Sample_12_0258 | Embryo | ESCs WT | 1103
Nanog | ZHBTc4 | Sample_12_0258 | Embryo | ESCs WT | 1042
Nanog | E14TG2a-4 | Sample_12_0258 | Embryo | ESCs WT | 961
SE ID | SE Region | TF | TF binding site
SE_12_025800001 | chrX:169978502-170022673 | Nanog | chrX:169980410-169981238,
SE_12_025800002 | chr6:125403551-125540718 | Nanog | chr6:125403724-125404044,
SE_12_025800003 | chr17:70736634-70925796 | Nanog | chr17:70737460-70737769
TF | Sample ID | Biosample type | Tissue type | Biosample name | SE number | Details
Nanog | Sample_12_0128 | Stem cell | Embryo | E14Tg2a treat 6h | 448
Nanog | Sample_12_0129 | Stem cell | Embryo | E14Tg2a treat 12h | 421
Nanog | Sample_12_0258 | Stem cell | Embryo | ESCs WT | 413
SE ID | TF | Region | Score | PValue | Seq
SE_12_025800003 | Nanog | chr6:125425784-125425795(+) | 15.6269 | 4.63e-07 | TTAA...
SE_12_025800003 | Nanog | chr6:125425784-125425795(-) | 15.3881 | 6.66e-07 | TTTA...
SE_12_025800002 | Nanog | chr6:125425784-125425795(-) | 16.8421 | 4.93e-07 | GGGA...
D
SE associated network
Associated gene
Super-enhancer
TF
SE associated genes
SE
gene
Strategies/Algorithm
Closest
SE_12_025800003
Dlgap
PreSTIGE
Tgif1
Overlap
Closest active
Myl126
Overlap with other super-enhancers
Overlap rate | SE ID | SE Region | Species | Sample ID | Tissue type | Biosample name
100% | SE_12_012700001 | chr17:70707694-70925948 | Mouse | Sample_12_0127 | Embryo | E14Tg2a treat 1h
100% | SE_12_012800002 | chr17:70718181-70926443 | Mouse | Sample_12_0128 | Embryo | E14Tg2a treat 6h
100% | SE_12_074700004 | chr17:70720433-70925908 | Mouse | Sample_12_0747 | Embryo | MEF-derived iPS cells
E
Expression of Nanog
Tissue(Fantom5)
Tissue(ENCODE)
Primary cell(Fantom5)
700
600
500
400
300
200
100
0
embryonic.stem.cell
mesodermal.cell
cardiac.precursor
cardiomyocyte
M.cell
Sox2+ supporting.cells
T.cell
Disease information of Nanog
TF name | Disease name | Disease type | Disease class | Disease semantic type
Nanog | Embryonal Neoplasm | disease | C04 | Neoplastic Process
Nanog | Neoplasms, Germ Cell and Embryonal | group | C04 | Neoplastic Process
Nanog | Germ cell tumor | group | C04 | Neoplastic Process
Nanog | Neoplasms, Embryonal and Mixed | disease | C04 | Neoplastic Process
