# Supplementary material for: SEdb 2.0: a comprehensive super-enhancer database of human and mouse
Source: Nucleic Acids Res. 2022 Nov 1;51(D1):D280–90. doi: 10.1093/nar/gkac968 (PMC9825585; doi:10.1093/nar/gkac968)
Supplement: gkac968_Supplemental_Files [file gkac968_supplemental_files.zip › Supplementary Table1.docx]

**Supplementary Table 1.** Software and parameters used by SEdb 2.0 to identify super-enhancers.

| **Software** | **Parameter** |
| --- | --- |
| fasterq-dump (2.9.6) | fasterq-dump --split-3 *******.sra |
| Bowtie2 (2.4.4) | bowtie2 -p 12 -x ref_genome/bowtie2index -U *******.fastq -S *******.sam |
| Samtools (1.7) | samtools view -b -S *******.sam -o *******.bam  samtools sort *******.bam -o *******.sort.bam  samtools index *******.sort.bam *******.sort.bam.bai |
| MACS2 (2.2.7) | macs2 callpeak -t *******_cas.sort.bam -c *******_input.sort.bam -f BAM -B -g hs(mm)^a^ -n -q 0.01 |
| ROSE main.py | python ROSE_main.py -g HG38(MM10)^b^ -i *******.gff –c *******_input.sort.bam -r *******_cas.sort.bam -o ******* -s 12500 |

^a^ ‘hs’ corresponds to human and ‘mm’ corresponds to mouse.

^b^ ‘HG38’ corresponds to human and ‘MM10’ corresponds to mouse.
